# Transcription Factor Klf4, Induced in the Lung by Oxygen at Birth, Regulates Perinatal Fibroblast and Myofibroblast Differentiation

**DOI:** 10.1371/journal.pone.0054806

**Published:** 2013-01-23

**Authors:** Jyh-Chang Jean, Elizabeth George, Klaus H. Kaestner, Lou Ann Scism Brown, Avrum Spira, Martin Joyce-Brady

**Affiliations:** 1 The Pulmonary Center, Boston University School of Medicine, Boston, Massachusetts, United States of America; 2 College of Engineering, Bioinformatics Program, Boston University, Boston, Massachusetts, United States of America; 3 Section of Computational Biomedicine, Boston University School of Medicine, Boston, Massachusetts, United States of America; 4 Department of Genetics, University of Pennsylvania School of Medicine, Philadelphia, Pennsylvania, United States of America; 5 Department of Pediatrics, Emory University School of Medicine, Atlanta, Georgia, United States of America; University of Bergen, Norway

## Abstract

The fluid-filled lung exists in relative hypoxia *in utero* (∼25 mm Hg), but at birth fills with ambient air where the partial pressure of oxygen is ∼150 mm Hg. The impact of this change was studied in mouse lung with microarrays to analyze gene expression one day before, and 2, 6, 12 and 24 hours after birth into room air or 10% O_2_. The expression levels of >150 genes, representing transcriptional regulation, structure, apoptosis and antioxidants were altered 2 hrs after birth in room air but blunted or absent with birth in 10% O_2_. Kruppel-like factor 4 (Klf4), a regulator of cell growth arrest and differentiation, was the most significantly altered lung gene at birth. Its protein product was expressed in fibroblasts and airway epithelial cells. Klf4 mRNA was induced in lung fibroblasts exposed to hyperoxia and constitutive expression of Klf4 mRNA in Klf4-null fibroblasts induced mRNAs for p21^cip1/Waf1^, smooth muscle actin, type 1 collagen, fibronectin and tenascin C. In Klf4 perinatal null lung, p21^cip1/Waf1^mRNA expression was deficient prior to birth and associated with ongoing cell proliferation after birth; connective tissue gene expression was deficient around birth and smooth muscle actin protein expression was absent from myofibroblasts at tips of developing alveoli; p53, p21^cip1/Waf1^ and caspase-3 protein expression were widespread at birth suggesting excess apoptosis compared to normal lung. We propose that the changing oxygen environment at birth acts as a physiologic signal to induce lung Klf4 mRNA expression, which then regulates proliferation and apoptosis in fibroblasts and airway epithelial cells, and connective tissue gene expression and myofibroblast differentiation at the tips of developing alveoli.

## Introduction

The lung is subject to major environmental changes in oxygen tension at birth when there is a rapid replacement of fluid, which filled the lung *in utero,* with ambient air during the first few breaths [1]. Antioxidant gene induction in the fetal lung late in gestation is believed to protect against injury associated with this dramatic change in oxygen tension [2]–[4]. In addition, environmental stimuli at birth, like alveolar oxygen tension, may serve a physiologic signal to initiate expression of genes involved with development of the postnatal lung [1], [5].

To pursue this latter role, we used gene expression arrays to compare birth-related lung gene expression changes in late fetal and newborn mice delivered into room air and 10% oxygen. The gene with the most dramatic change at birth is transcription factor Kruppel-like factor 4. Klf4 regulates cellular proliferation and differentiation [6]–[8]. The critical role of Klf4 in epithelial cell differentiation was revealed by targeted deletion of the mouse Klf4 gene as barrier function in the epidermal layer of the skin was disrupted leading to dehydration and death within 15 hours after birth [9]. Epithelial cell proliferation in the skin was not affected, nor was apoptosis. This early postnatal lethality did not appear to involve loss of Klf4 expression from other organ systems [9]. In some tissues, this ability of Klf4 to regulate cell proliferation and differentiation is directly opposed by Klf5, another Kruppel-like family member. This competitive duel has been described specifically in intestinal epithelial cells and vascular smooth muscle cells [10] but it is not known to occur in the lung. However, conditional deletion of Klf5 from lung epithelial cells has revealed a critical role for Klf5 in perinatal lung development as its loss produced a lethal phenotype at birth with impaired alveolar epithelial cell maturation, surfactant deficiency and insufficiency of alveolar gas exchange surface. Lung epithelial cell proliferation was not affected [11]. Since Klf4 is known to function as a regulator of cell proliferation and differentiation and to be expressed in the normal fetal and newborn lung [6], [7], we hypothesized that it responds to the changing oxygen environment at birth to regulate these cellular processes given that Klf4 messenger RNA changed so dramatically in the lung at birth.

In this study we use microarrays to show that birth into 21% oxygen is associated with a change in the level of expression of many genes in the lung representing transcriptional regulation, structure, apoptosis and antioxidant activity. These changes in the lung are dramatically attenuated with birth into 10% oxygen, and are not seen in the liver at birth in room air, suggesting that the changes are in response to the rise in alveolar oxygen. Transcription factor Klf4 is the gene that exhibited the greatest change in perinatal lung gene expression. Its messenger RNA is induced 5-fold in vivo and we show that it can respond to hyperoxia in lung fibroblasts at a transcriptional level in vitro, independent of protein synthesis. While Klf4 protein is expressed in fibroblasts and epithelial cells, we show that Klf4 expression is required for fibroblast function in vivo using lung tissue and fibroblasts from the Klf4 null mouse. In the absence of Klf4, messenger RNA for p21^cip1/Waf1^, a mediator of Klf4 growth arrest activity [10], is decreased in the fetal lung at day 19 of gestation and lung cells continue to proliferate at birth. Connective tissue genes expressed by fibroblasts are Klf4 regulated. These include smooth muscle actin, fibronectin, tensacin C and type 1 collagen. With loss of Klf4 expression, their level of mRNA expression is decreased in the fetal lung and the lung at birth, specifically in lung myofibroblasts. Klf4 also inhibits p53 mediated apoptosis [12]. Klf4 absence is associated with excess apoptosis in fibroblasts and epithelial cells in the Klf4 null lung at birth compared to normal lung, assessed by nuclear expression of p53, p21^cip1/Waf1^ and caspase-3. The change in alveolar oxygen at birth appears to serve as a signal that activates gene expression at birth, particularly Klf4 mRNA expression. Loss of this growth arrest and differentiation-related gene leads to persistent cell proliferation, excessive apoptosis, and impaired fibroblast differentiation in the perinatal lung.

## Materials and Methods

### Ethics Statement

This study was performed in strict accordance with the recommendations in the Guide for the Care and Use of Laboratory Animals on the National Institutes of Health. The Institutional Animal Care and Use Committee of Boston University School of Medicine approved this protocol (Protocol number AN-14284). All surgery was performed under sodium pentobarbital anesthesia, and all efforts were made to minimize suffering.

### Mouse Protocol

Timed-pregnant C57B/6J dams were obtained from Jackson Laboratories (Bar Harbor, ME) at day 15 of gestation and individually housed in a constant temperature and humidity environment with a 12∶12 light: dark cycle. Some were sacrificed six days later to harvest lung and liver at the end of gestation (day 21). Others were monitored until parturition and newborn lung and liver were harvested at 2, 6, 12 and 24 hours after birth. A separate group of pregnant dams were placed in a 10% oxygen environment using a glove bag six days after arrival from Jackson Laboratories [13]. Some dams were sacrificed after 12 hours in 10% oxygen, and lung tissue was harvested from fetuses to assess the status of baseline fetal measures at the end of gestation (day 21), while other dams were monitored until parturition and newborn tissues samples were obtained at the same times as above. All dams, fetuses and newborns survived in 10% oxygen. Mice were sacrificed using a lethal intra-peritoneal dose of sodium pentobarbital, and rapidly dissected to obtain lung and liver samples.

### Microarray Studies

Total RNA was harvested from snap frozen tissue samples (n = 3 for each time point), processed, and hybridized to Affymetrix MGU74Av2 arrays [14], [15]. Microarray data were analyzed using Affymetrix Microarray Suite 5.0 software (MAS5.0), and target intensity for each chip was set to 100 for normalization. All chips had detection p values less than 0.05 for approximately 40% of probes with Gapdh 3′:5′ ratio of ∼1. The detection p-value defined by MAS5.0 was used as a quality control filter and only genes with median detection p<0.05 across all samples were considered for further statistical and computational analyses. Information about genes on the array can be found on the Affymetrix website (http://www.affymetrix.com/analysis/downloadcenter.affx). A two-sample t-test (p<0.01) was used to identify genes differentially expressed between the baseline level of expression just prior to birth (fetal day 21, F21) and each of the time points after birth. The potential for a type 2 error exists due to the presence of multiple comparisons in array data. Current methods to adjust for multiple comparisons, such as Bonferroni correction, are too conservative and break down quickly in the setting of microarrays [16]. The Bonferroni correction also assumes independence of the different tests, which is unlikely to hold true in the microarray setting where multiple genes are co-regulated. Our p<0.01 threshold was selected to limit the false discovery rate, and we compared the number of genes at this threshold with the expected number of false positives under the null hypothesis so as to estimate the overabundance of information in the analyzed dataset [17]. We recognize that there may still be false positives in our gene sets, so genes of interest, identified as differentially expressed by microarray, were confirmed experimentally. Hierarchical clustering was performed on genes that changed between F21 and 2 h and 6 h after birth lung in room air using CLUSTER and TREEVIEW programs (http://rana.lbl.gov/EisenSoftware.htm). The analysis was performed with normalized, median-centered data using an un-centered Pearson correlation similarity metric and average linkage clustering. Functional categories that were overrepresented among these genes were identified using GoMiner [18] and EASE [19]. Microarray data are deposited in the NCBIs Gene Expression Omnibus (GEO, http://www.ncbi.nlm.nih.gov/geo/), and are accessible through GEO Series accession number GSE4310.

### Klf4 Validation Studies

Microarray results for Klf4 were confirmed by Northern analysis, normalized to 18S by quantifying absolute counts on a PhosphorImager [20], [21], and immunohistochemistry with a commercial antibody against Klf4 (Santa Cruz Biotechnology, Santa Cruz, CA) using standard methodology in paraffin-embedded tissues [22], [23]. The effect of oxygen on Klf4 mRNA expression was determined by exposing the newborn mouse mesenchymal cell line MLg to an atmosphere of 95% oxygen in modular incubator chambers as described [5], [24]. MLg cells were obtained form ATCC (catalogue CCL-206). Actinomycin D and cycloheximide were added to hyperoxia-exposed MLg cells to determine if Klf4 messenger RNA induction was affected by synthesis of RNA or protein, respectively. Glutathione disulfide (GSSG) was measured in tissues by HPLC to determine if oxidant stress was present in perinatal tissues and expressed as nmol/mg tissue as described previously [13].

#### Analysis of Klf4 null mouse lung fibroblasts and lung tissue

Lung tissue from Klf4 null mice were obtained from the laboratory of Dr. Klaus H. Kaestner [25]. Klf4 null fibroblast cells (Fib-Klf4) were produced in The Pulmonary Center laboratory by selecting from a mixed population of trypsin-dispersed Klf4 null lung cells transfected with the pTargeT expression vector from Promega Corporation (Madison, WI) containing an SV40TAg cDNA insert and the neomycin phosphotransferase gene. Cells were passaged, isolated and then characterized for smooth muscle actin, type 1 collagen, vimentin, surfactant protein-C, cytokeratin 8 messenger RNA expression by Northern blot analysis. Murine lung epithelial (MLE) cells (ATCC catalogue CRL-2110) served as the control for the epithelial cell phenotype and MLg cells served as the control for the fibroblast phenotype [26].

To identify Klf4 target genes, the pTargeT vector contained a Klf4 cDNA insert was transfected into these Klf4 null cells to generate Fib+Klf4 cells. These Fib+Klf4 cells were selected for stable expression of Klf4 and assayed for messenger RNA expression of p21^cip1/Waf1^, smooth muscle actin, fibronectin, tenascin C, type 1 collagen and fibronectin receptor beta (Itgb1, NM_010578) by Northern analysis in two independent experiments. Cells expressing empty vector were selected separately to serve as the negative control.

To determine the impact of Kfl4 deficiency on Klf4 target gene expression in vivo, lung tissue samples from Klf4 null mice (n = 3), and matched wild type littermate controls were also obtained from Dr. Kaestner’s laboratory at day 19 of gestation (F19) and 6 hours after birth in room air [25]. Tissue was immediately processed and transferred to the Pulmonary Center at Boston University School of Medicine for analysis. Gene expression in Klf4 null lung and normal lung was assessed by real time PCR using primers from the Applied Biosystems (Carlsbad, CA) website as described [23]. Paraformaldehyde-fixed tissue was dehydrated, embedded and sectioned for histological analyses in The Pulmonary Center laboratory of Dr. Joyce-Brady (Boston, MA).

#### Cell proliferation, apoptosis and smooth muscle actin protein expression 6 h after birth

Cellular proliferation was assessed by immunohistochemistry using antibodies against proliferating cell nuclear antigen (PCNA, FL-261, sc-7907). Cellular apoptosis was assessed by immunohistochemistry using antibodies against activate phosphorylated p53 (sc-18078-R, mSer20), p21^cip1/Waf1^ (C-19 sc-397) and caspase-3 (H-277, sc-7148) [27]. All antibodies were from Santa Cruz Biotechnology (Santa Cruz, CA). Messenger RNA expression for p21^cip1/Waf1^ was also assessed using real time PCR. Smooth muscle actin was localized with an alpha-smooth muscle actin monoclonal antibody (#A2547) from Sigma (St. Louis, MO) and a secondary fluorescent donkey anti-mouse antibody (Alexa Fluor 488: A21202) from Invitrogen (Grand Island, NY). All immunohistochemistry employed standard methodology with positive and negative controls as described previously [13], [27].

#### Statistics

Nominal data are presented as means and standard error from n = 3 experiments. Data were compared by ANOVA and Tukey’s honestly significant test for post hoc comparison of means using Statistica software package from StatSoft (Tulsa, OK). P<0.05 was considered significant.

## Results

### Transcriptional Profile of Gene Expression in the Lung at Birth

**Figure 1:**
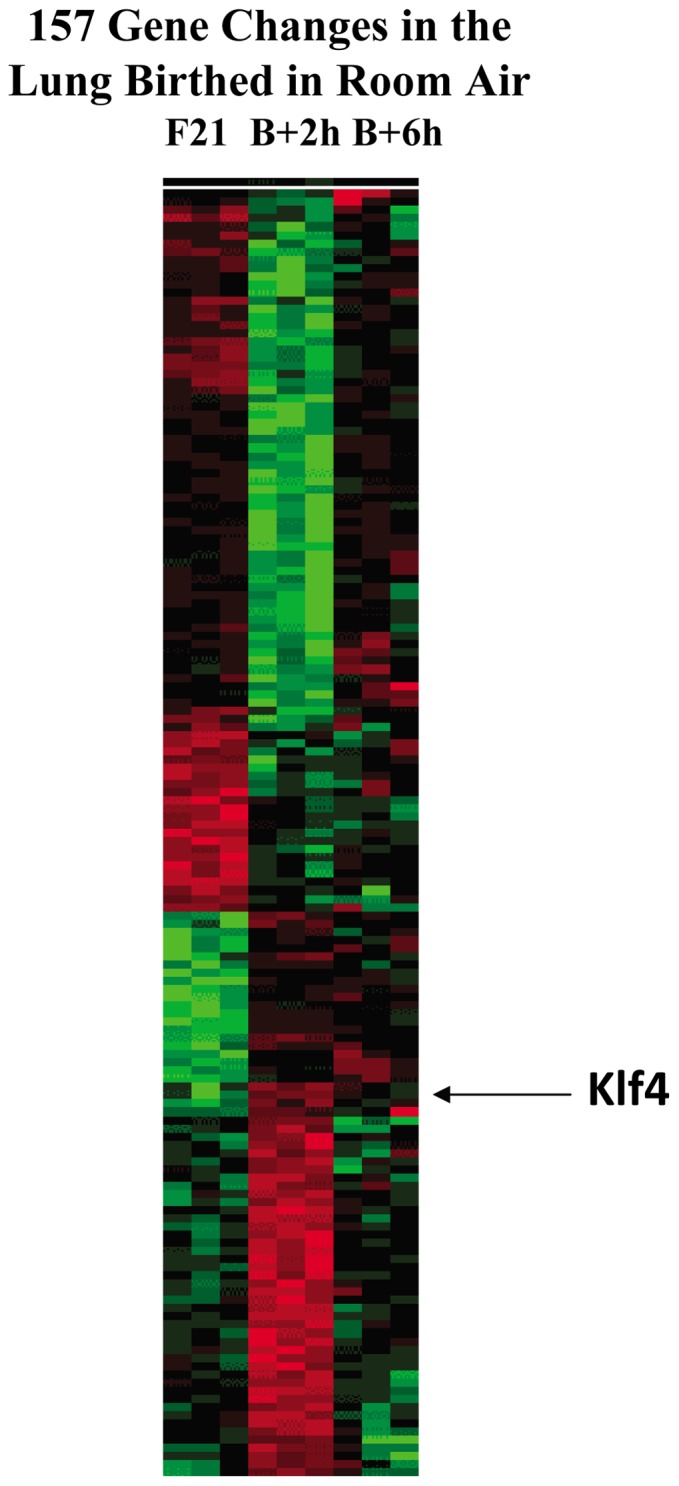
Transcriptional profile of gene expression in lung at birth in room air. Heirarchical cluster of the 157 genes that were up-regulated (red) or down-regulated (green) after birth (B) in room air at 2 h (B +2) and 6 h (B +6) compared to one day prior to birth on the last day of gestation (fetal day 21: F21). Four patterns are evident: transient up or down regulation and persistent up or down regulation. Arrow denotes Kruppel-like factor 4 (Klf4), a transcriptional regulator that exhibited the highest t-test (p<0.00001) and the greatest fold change at birth (5-fold).

**Figure 2:**
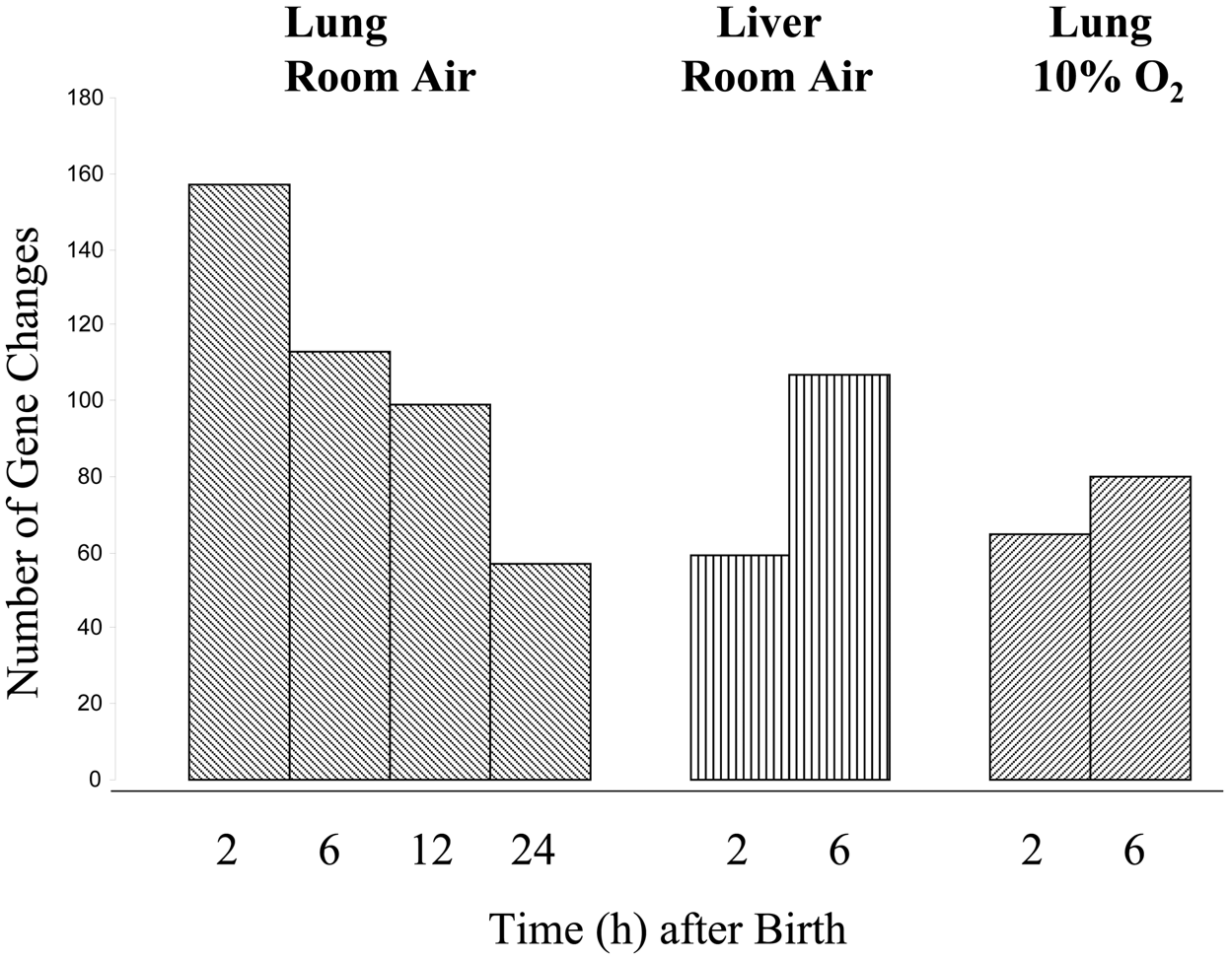
Number of genes changing their level of expression at birth. In the lung birthed in room, the greatest number of gene changes (157) was at 2 h and transcriptional regulation, structure, apoptosis and antioxidant activity were overrepresented functional categories. Thereafter the numbers declined to 113, 99 and 57 at 6 h, 12 h and 24 h, respectively. In the liver birthed in room air, there were only 59 gene changes at 2 h and 100 at 6 hr. In the lung birthed into 10% oxygen, there were only 65 gene changes at 2 h and 80 at 6 h. No functional category of gene expression was overrepresented in either dataset.

Informative expression signals were obtained from approximately 30% of the probes on the microarray, as measured by significant median detection p-value across all samples. Comparison of lung expression values at 21 days of gestation (F21) and with each time point after birth into room air revealed that the expression of 157 genes was significantly altered at 2 h (p<0.01). Only 40 gene changes were expected by chance alone at this level of significance (**Figure 1**). The major functional categories overrepresented in the 157 gene changes were transcriptional regulation, structure, apoptosis and antioxidant activity. After 2 h, there was a steady decline in the number of gene changes to 113, 99 and 57 at 6 h, 12 h and 24 h, respectively. Birth into 10% oxygen decreased the number of gene changes to only 65 at 2 h and 80 at 6 h, and eliminated overrepresentation of any functional category of gene expression. In comparison, the total number of gene changes in the liver birthed in room air was only 59 at 2 h and 100 at 6 h (**Figure 2**). No functional category of gene expression was overrepresented in the liver microarray data.

After birth in room air, transcription factor Klf4 was the gene with the most significant change by t-test in the lung (p<10^−5^) and it increased by 5-fold. Klf4 mRNA expression did not change in the lung birthed in 10% oxygen, nor in the liver birthed in room air.

### Confirmation of Klf4 mRNA Induction in Normal Lung at Birth

**Figure 3:**
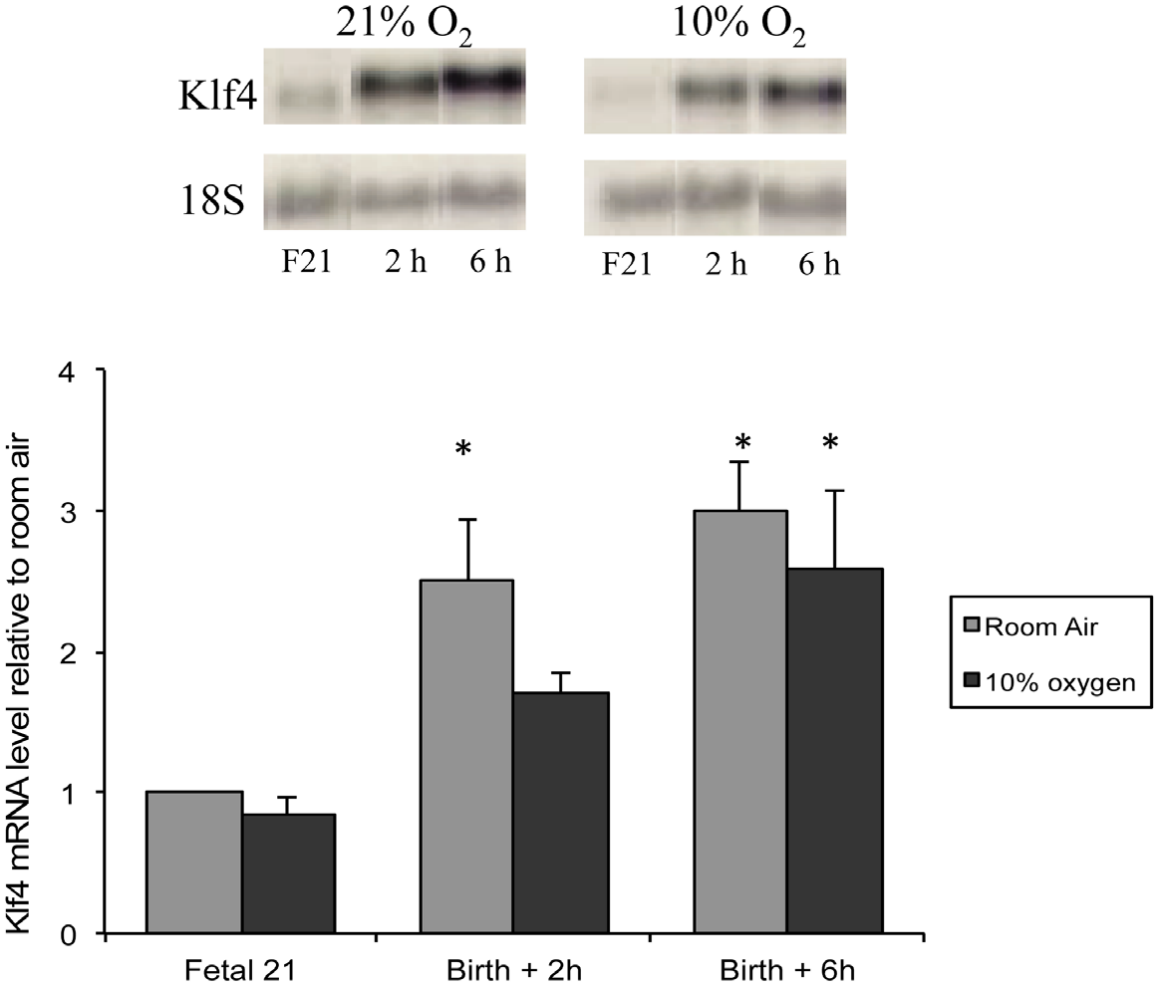
Confirmation of Klf4 mRNA Induction in the Lung at Birth. Klf4 mRNA induction was confirmed by Northern analysis (2.5 fold at 2 h and 3-fold at 6 h in 21% oxygen, n = 3, p<0.05). Klf4 mRNA induction at 2 h was attenuated by birth in 10% oxygen (1.7 fold, n = 3, p>0.05) and a shift of the induction response to the right. Asterisks denote significant increase above that of F21 lung in room air.

**Figure 4:**
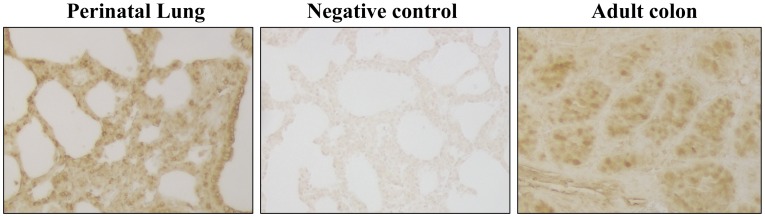
Klf4 Immunohistochemistry. Klf4 protein is detected within nuclei of interstitial cells and airway epithelial cells of perinatal lung in the left panel. Signal is absent with omission of primary antibody as a negative control in the middle panel. Signal is present in epithelial cell nuclei of adult colon as a positive control in the right panel.

**Figure 5:**
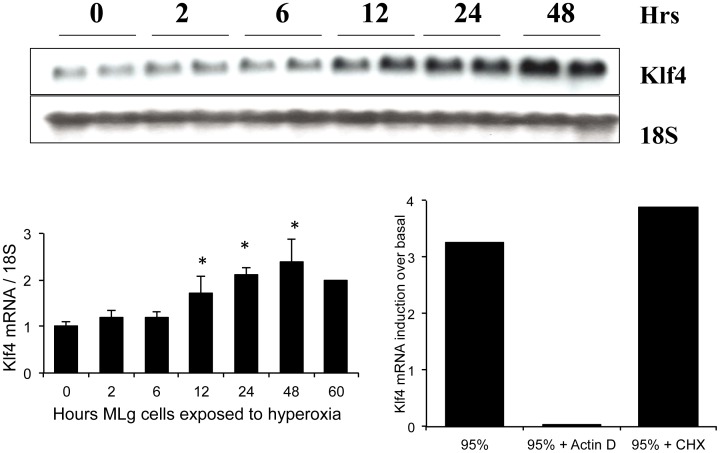
Regulation of fibroblast Klf4 messenger RNA expression by oxygen. Exposure of mouse lung fibroblasts (MLg cells) to 95% oxygen induces Klf4 mRNA by 2.1 fold after 12 h (n = 4, p<0.05) and 2.8-fold by 48 h (n = 4, p<0.05). Klf4 mRNA induction in MLg cells exposed to 95% oxygen is blocked by pre-treating the cells with actinomycin D (95%+Actin D), but not cycloheximide (95%+CHX). Asterisks denote p<0.05.

**Figure 6:**
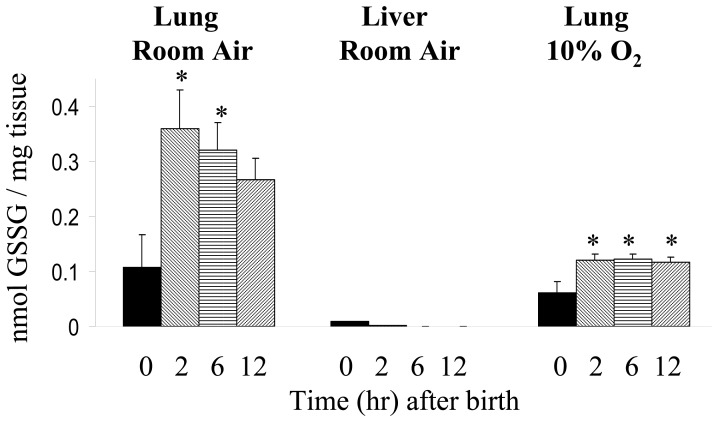
Glutathione disulfide accumulation at birth. Glutathione disulfide (GSSG) was measured in the lung and the liver of mice just before birth (time 0) and then 2 h, 6 h and 12 h after birth in room air and at similar times in the lungs of mice born in 10% oxygen. Asterisks denote p<0.05 compared to value at time 0.

Northern blot analysis confirmed our microarray data of lung Klf4 mRNA induction in room air at 2 h (3.5 fold, **Figure 3**). Klf4 protein was localized to epithelial cells in the adult colon by immunohistochemistry, but to lung interstitial cells and airway epithelial cells in the perinatal lung (**Figure 4**). Klf4 messenger RNA localized to colonic epithelial cells in the adult mouse by *in situ* hybridization analysis but the signal was diffuse in perinatal mouse lung, predominating in the mesenchyme and not the airway epithelium (data not shown). Since airway epithelial cells alone could not have accounted for the induction of Klf4 messenger RNA in the lung at birth, we focused our study on fibroblasts, a major component of the mesenchyme. To determine if fibroblast Klf4 mRNA expression responds to hyperoxia, we exposed lung fibroblasts (MLg cells) to 95% oxygen and found that Klf4 mRNA increased 2 fold at 12 h and peaked at 2.5-fold at 48 hours. Klf4 mRNA induction in hyperoxia was blocked if MLg cells were pretreated with actinomycin D, but not cycloheximide, suggesting transcriptional regulation in hyperoxia that was not dependent on protein synthesis (**Figure 5** **)**. Since hyperoxia induces oxidant stress, we measured glutathione disulfide in whole lung tissue to determine if oxidant stress was present in the lung with birth in room air (**Figure 6**). In the fetal lung at day 21 of gestation, GSSG content began at 0.107±0.06 nmol/mg tissue and rose abruptly to 0.359±0.07 nmol/mg lung tissue at 2 h after birth (3.5 fold, n = 3, p<0.05), then declined to 0.320±0.05 nmol/mg lung tissue at 6 h (2.9-fold, n = 3) and 0.266±0.04 nmol/mg lung tissue at 12 h (2.5-fold, n = 3) after birth. In 10% oxygen, GSSG content began at 0.062 nmol/mg lung tissue at day 21 of gestation, rose to only 0.12 nmol/mg lung tissue at 2 h (2-fold, n = 3, p<0.05) after birth, and remained at this level at 6 h (n = 3) and 12 h (n = 3) after birth. In room air, GSSG content began at only 0.009±0.001 nmol/mg tissue in fetal liver at day 21 of gestation, and did not rise at all. Rather it declined at 2 (n = 3), 6 (n = 3) and 12 h (n = 3) after birth.

### Lung Connective Tissue Genes are Klf4 Targets

**Figure 7:**
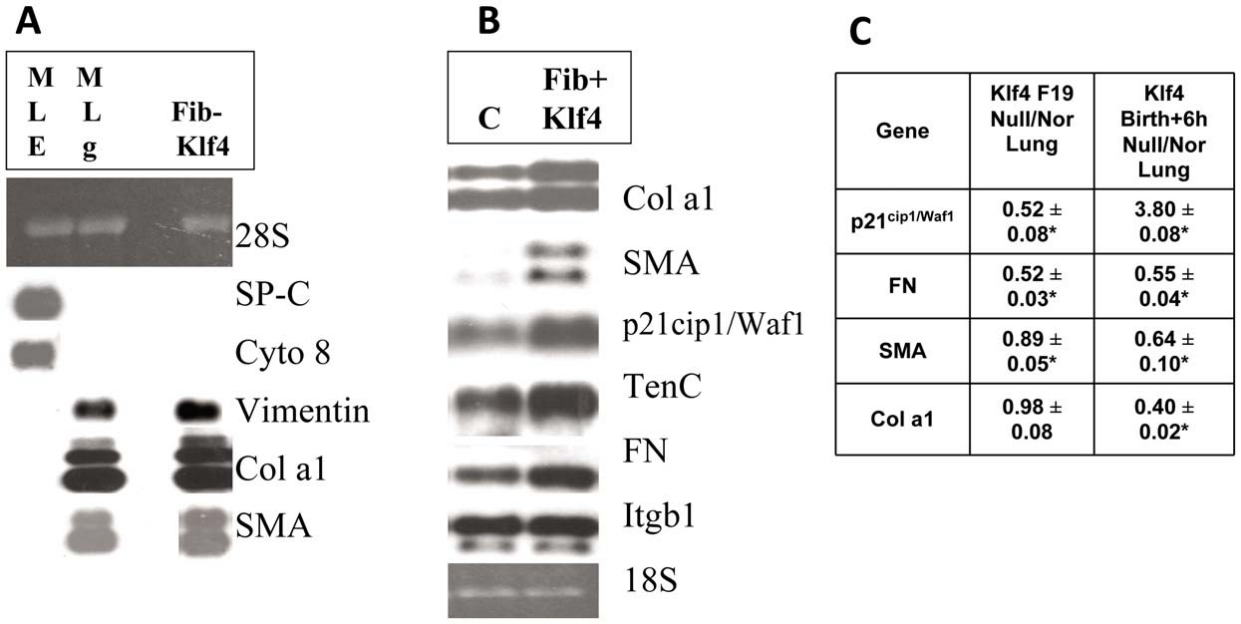
Decrease in connective tissue gene expression in perinatal lung with Klf4 deficiency. (**A**) Cells from the Klf4 null lung were immortalized with SV40Tag as described in Methods. These Klf4 null fibroblasts (Fib-Klf4) express vimentin, the alpha 1 chain of Type 1 collagen (Col a1) and smooth muscle actin (SMA) in common with the mouse lung fibroblasts (MLg), but neither SP-C nor cytokeratin 8 which are found in mouse lung epithelial cells (MLE). The ribosomal marker 28S is shown as a loading control. (**B**) Klf4 cDNA was constitutively expressed in Fib-Klf4 cells to generate Fib+Klf4 cells as described in Methods. Compared to the empty vector control cells (C), these Klf4 expressing fibroblasts (Fib+Klf4) exhibit up-regulated expression of messenger RNA for p21^cip1/Waf1^ (2 fold), SMA (3.5 fold), the alpha 1 chain of Type 1 collagen, tenascin C and fibronectin (2–2.5 fold each) but not fibronectin receptor beta (Itgb1). The ribosomal marker 18S is shown as a loading control. These data represent two independent Northern blot experiments. (**C**) Comparative levels of expression for these Klf4 target genes in Klf4 null lung (null) relative to normal lung (Nor) at fetal day 19 of gestation and 6 h after birth in room air (n = 3 for each sample, asterisk denotes difference in level of expression in Klf4 null lung versus normal lung at p<0.05).

To identify Klf4 target genes in lung fibroblasts we first immortalized and characterized a Klf4 null lung cell line as described in Methods. These cells where fibroblasts as they expressed messenger RNAs for vimentin, the alpha 1 chain of type I collagen and smooth muscle actin in common with MLg cells, but neither SP-C nor cytokeratin, epithelial markers found in MLE cells (Fib-Klf4, **Figure 7A**). Klf4 target genes were assessed after transfecting these cells with an expression vector containing full length Klf4 cDNA and selecting for stable expression. Klf4 expression in these fibroblasts, Fib+Klf4 cells, induced expression of p21^cip1/Waf1^ by 2.1 fold, smooth muscle actin by 3.5 fold, and the alpha 1 chain of Type I collagen, fibronectin and tenascin C by 2–2.5 fold compared to that of control cells (C), which stably expressed empty vector alone. Fibronectin receptor beta (Itgb1) messenger RNA expression and 18S ribosomal RNA expression were unchanged between the two cell types as a negative control and a loading control, respectively. These data represent two independent Northern blot experiments (**7B**). To determine the impact of Klf4 deficiency on expression of these genes *in vivo*, Klf4 null fetal lung was examined at day 19 of gestation and compared to normal fetal lung (**7C**). The level of gene expression in the Klf4 null lung as a percentage of normal was 52±8% for p21^cip1/Waf1^ (n = 3, p<0.05), 52±3% for fibronectin (FN, n = 3, p<0.05), 89±5% for smooth muscle actin (SMA, n = 3, p<0.05) and 98±8% for the alpha 1 chain of type 1 collagen (Col a1, n = 3).

### Klf4 Deficiency Impacts Proliferation, Apoptosis and Connective Tissue Gene Expression at Birth

**Figure 8:**
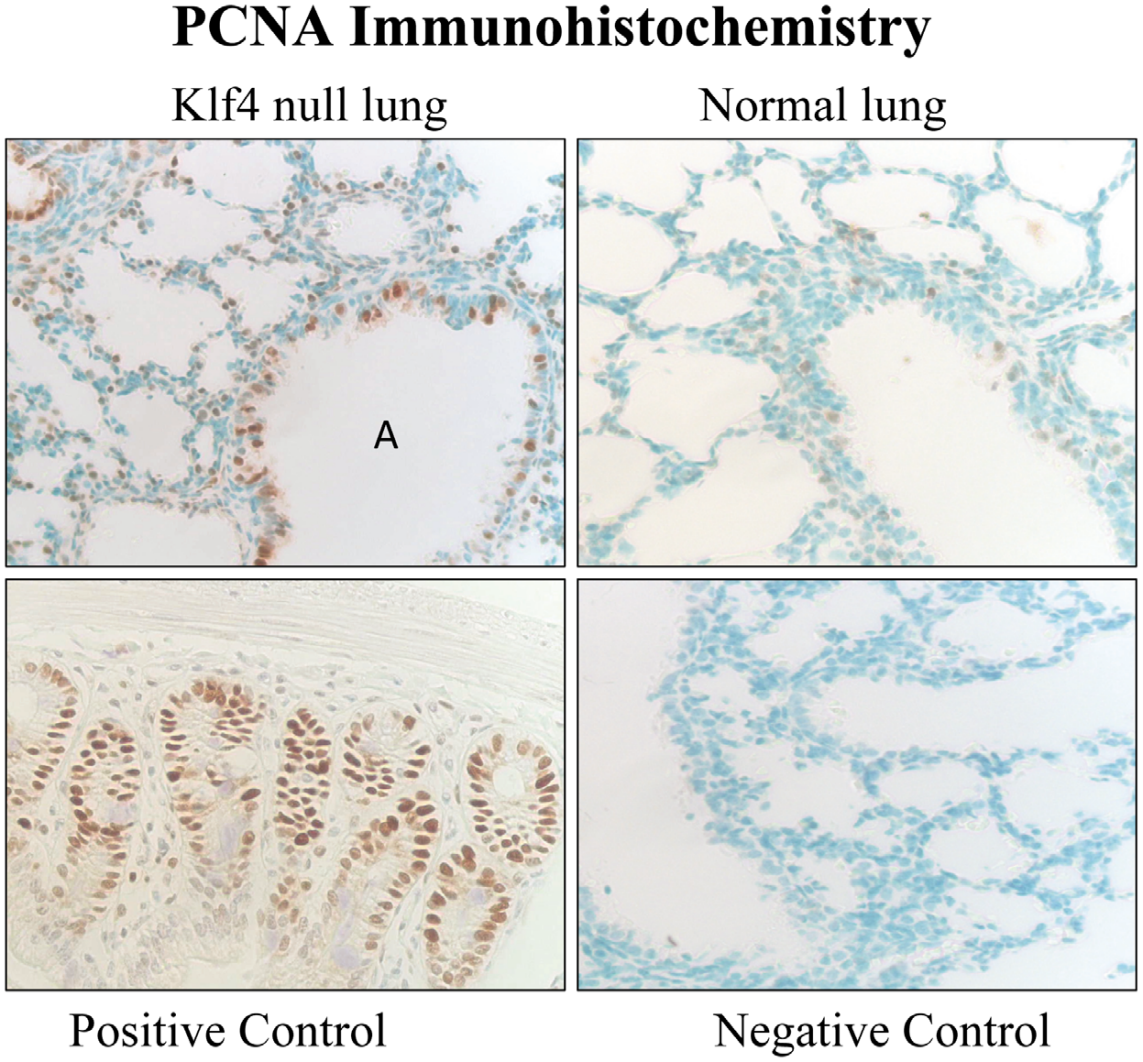
Proliferating cell nuclear antigen (PCNA) immunohistochemistry. An intense immunohistochemical signal (brown color) for PCNA protein is present in numerous mesenchymal and airway epithelial (A) cell nuclei from Klf4 null mouse lung (top left panel). Few cells of the normal lung exhibit any PCNA signal (top right panel). PCNA signal is present in proliferating epithelial cells of the colon as a positive control (bottom left panel). PCNA signal is absent with omission of primary antibody as negative control (bottom right panel).

Proliferation was assessed by nuclear expression of PCNA. At 6 h after birth a strong PCNA signal was present in many mesenchymal and airway epithelial cells in the Klf4 null lung suggesting ongoing cell proliferation. Few nuclei of the normal mouse lung at birth showed any PCNA signal. The positive control cell proliferation was epithelial cells from the crypts of the adult colon. Omission of primary antibody from Klf4 null lung samples served as the negative control **(** **Figure 8** **)**.

**Figure 9:**
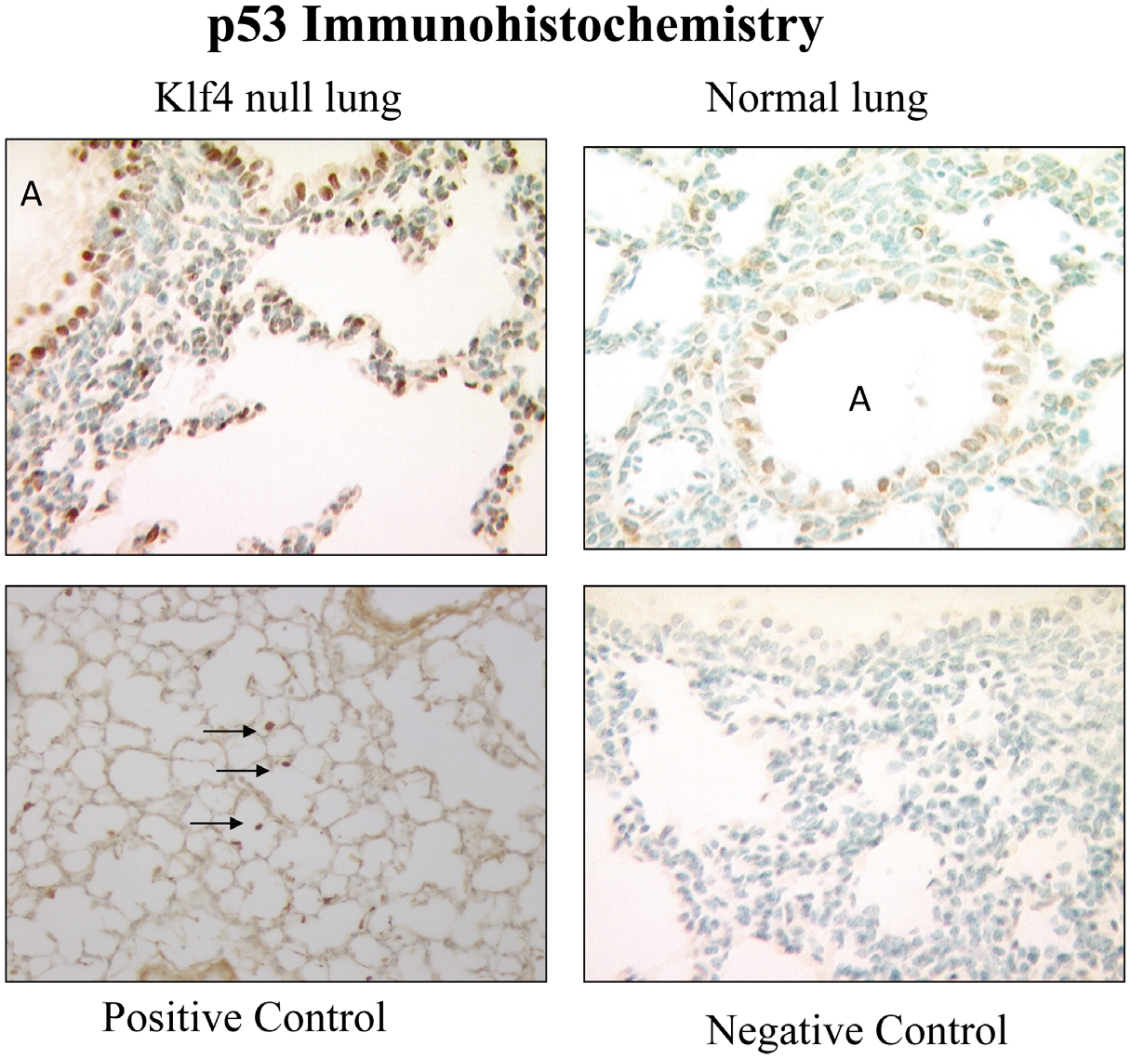
Nuclear localization of p53 protein. An intense immuno-histochemical signal (brown color) for p53 protein is present in numerous airway epithelial (A) and mesenchymal cells of newborn Klf4 null mouse lung (top left panel). Few cells in normal lung exhibit any signal (top right panel). Signal is present in macrophages of the hyperoxia-exposed GGT^enu1^ mouse lung (arrows) as a positive control [13] (bottom left panel). No signal is present with omission of primary antibody as a negative control (bottom right panel).

**Figure 10:**
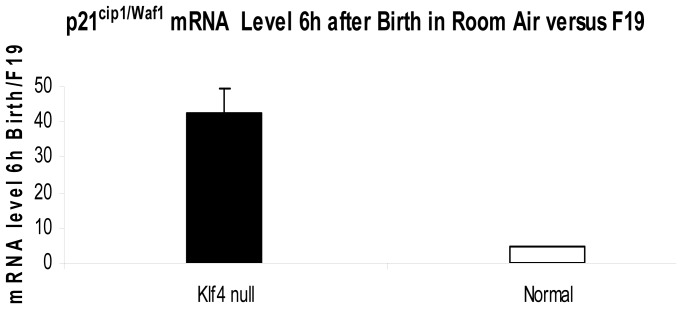
Induction of p21^cip1/Waf1^ messenger RNA and protein in Klf4 null lung versus normal lung at birth. The content of p21^cip1/Waf1^ mRNA increased 38-fold in Klf4 knockout lung between day 19 of gestation and 6 h after birth, but only 5-fold in normal lung.

**Figure 11:**
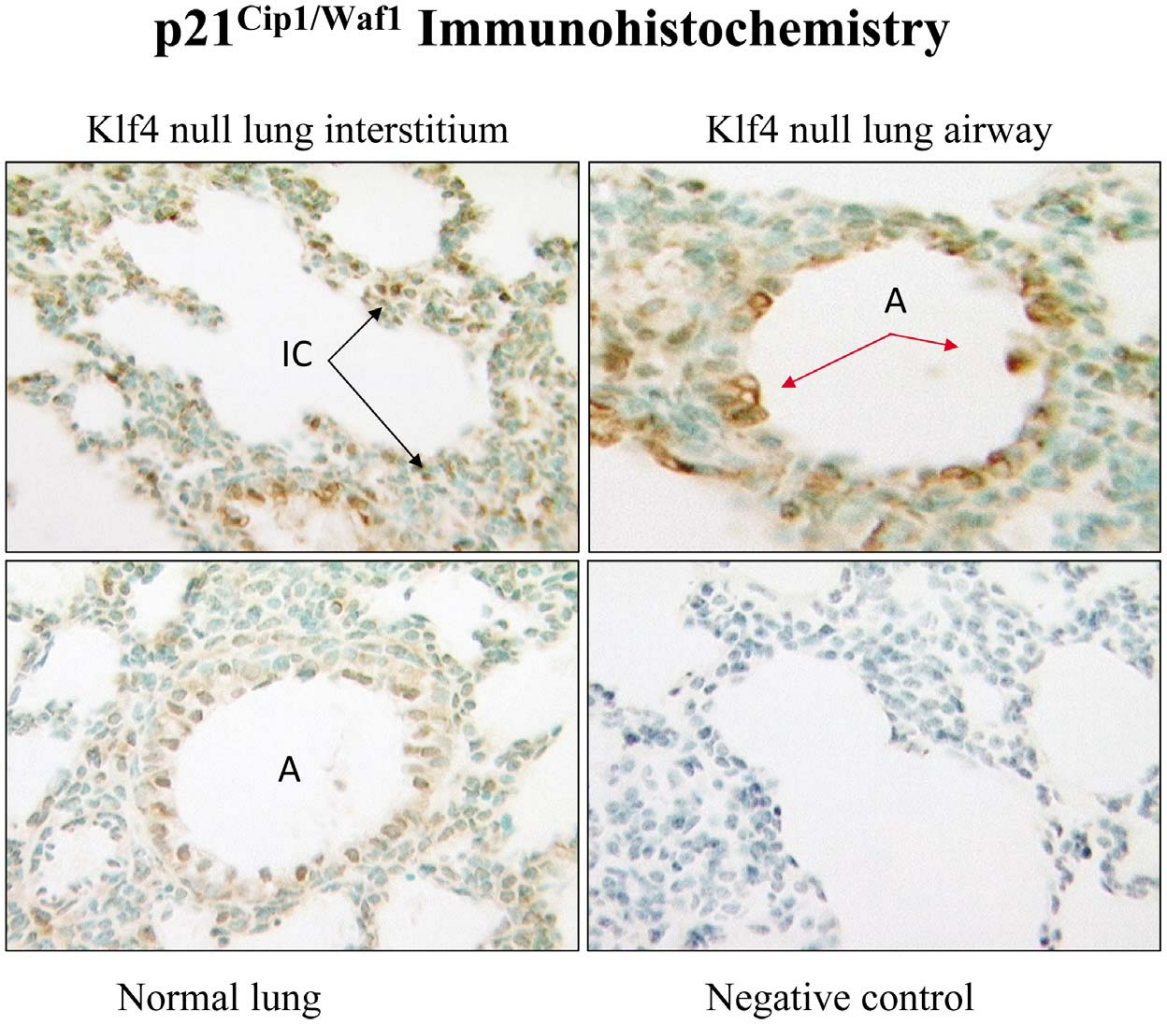
Immunohistochemisry for p21^cip1/Waf1^ protein. An intense nuclear immunohistochemical signal (brown color) for p21^cip1/Waf1^ protein is present in numerous interstitial (IC) cells (top left panel) and airway epithelial (A) cells (top right panel) of Klf4 null lung at birth. Signal is sparse in normal lung (bottom left panel) and absent with omission of primary antibody as the negative control (bottom right panel).

**Figure 12:**
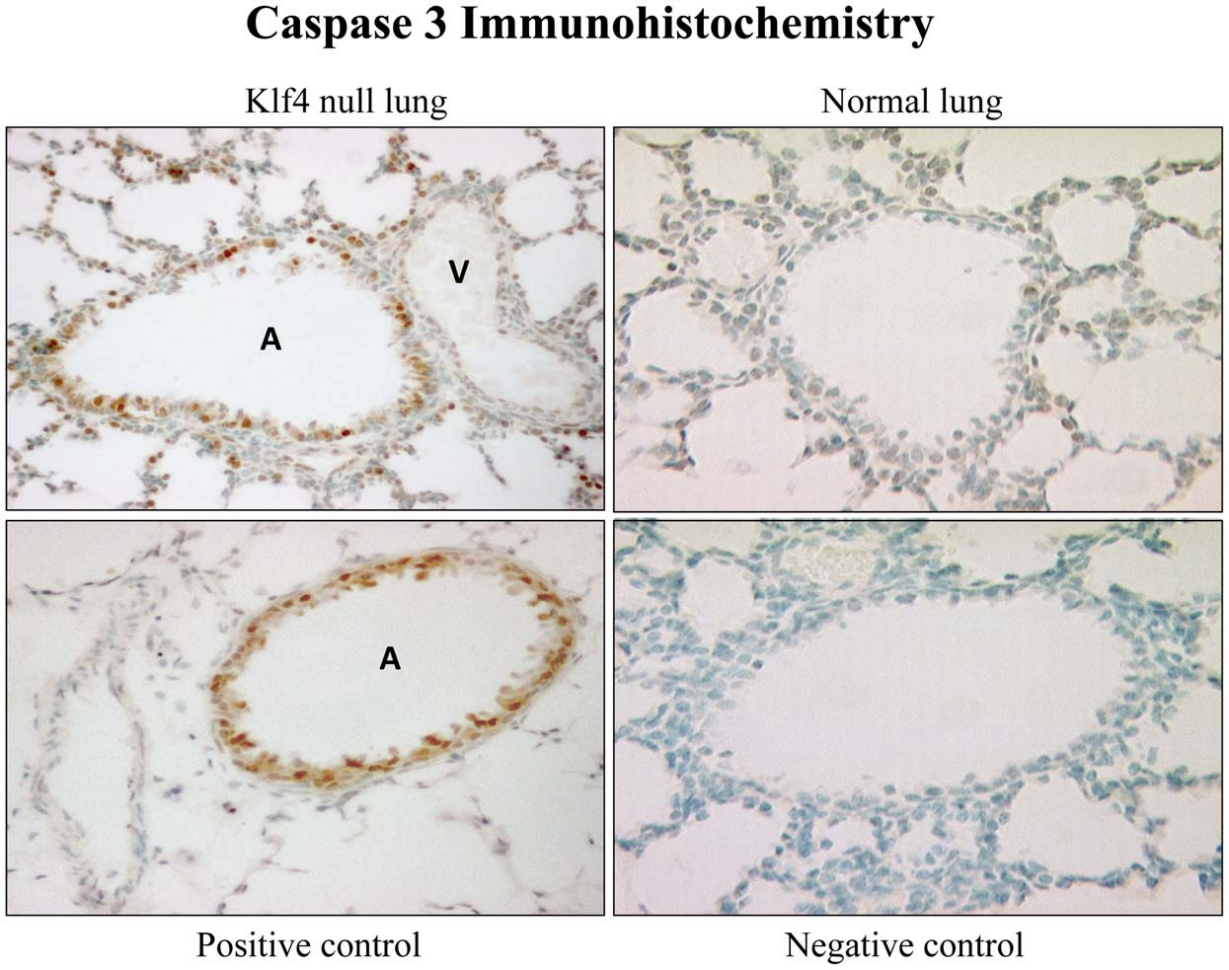
Lung cell apoptosis at birth. An intense nuclear immunohistochemical signal (brown color) for caspase-3 protein is present in numerous airway epithelial (A) and surrounding interstitial cells but absent from vascular (V) cells in Klf4 null mouse lung (top left panel). Few cells (arrow) of the normal lung exhibit signal (top right panel). Signal limited to airway epithelial (A) cells in hyperoxia-exposed GGT^enu1^ lung as a positive control [13] (bottom left panel). No signal with omission of primary antibody as the negative control (bottom right panel).

The presence of apoptosis was assessed with expression of p53, p21^cip1/Waf1^, and caspase-3. At 6 h after birth, the Klf4 null lung had prevalent nuclear signal for activated p53 in fibroblasts and airway epithelial cells **(** **Figure 9** **)**. Since p21^cip1/Waf1^ is a target of p53, we assessed its messenger RNA expression by real time PCR and protein expression by immunohistochemistry. At 6 h after birth, p21^cip1/Waf1^ mRNA expression in the Klf4 null lung became more abundant than that of normal lung by 3.8 fold. Overall this represents a 38-fold induction in the Klf4 null lung since p21^cip1/Waf1^ expression was induced 5-fold in normal lung between fetal day 19 of gestation and 6 h after birth, and began at 2-fold below that of normal lung at fetal day 19 (**Figure 10**). The immunohistochemical signal for p21^cip1/Waf1^ protein showed an intense and prevalent nuclear signal in many fibroblast and epithelial cells of the Klf4 null lung at birth. The corresponding signal was weak and sparse in normal lung cells (**Figure 11**). The presence of apoptosis was determined by nuclear localization of caspase-3. Numerous airway epithelial cells and mesenchymal cells expressed a nuclear caspase-3 signal. However, signal was not evident in vascular cells from large blood vessels, suggesting that apoptosis in the Klf4 null lung was not generalized at birth. Only a few cells from the normal lung exhibited a caspase-3 signal **(** **Figure 12** **).**

**Figure 13:**
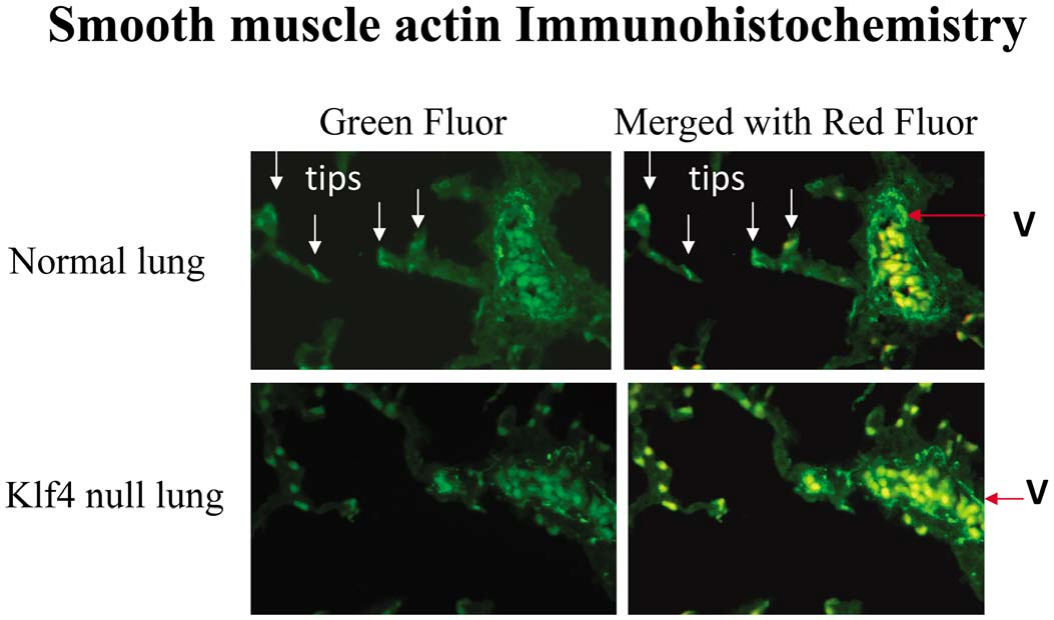
Immunohistochemistry for smooth muscle actin in lung at birth. In the normal lung at 6 hours after birth, a specific smooth muscle actin (SMA) signal (green) is present in myofibroblasts at the saccular tips (tips) and vascular smooth muscle cells from a large blood vessel (V) in the left panel (green fluorescence) and the right panel (green despite merger with red autofluorescence). Nonspecific yellow signal (green merged with red autofluoresecence) is present in hematopoietic cells in large blood vessels. In the Klf4 null lung, however, the specific green SMA signal is only present in vascular smooth muscle cells from a large blood vessel (V). Cells in saccular tips in the left panel are yellow after merger with red autofluorescent signal in the right panel denoting non-specific signal likely originating from hematopoietic cells in small blood vessels within the lung interstitium as in large blood vessels.

By 6 hours after birth, the relative expression of fibronectin mRNA in the Klf4 null lung remained only 55±4% (n = 3, p<0.05) of that of normal lung, whereas collagen mRNA showed a decline to only 40±2% (n = 3, p<0.05) of normal, and smooth muscle actin showed a decline to 64±10% (n = 3, p<0.05) of normal **(** **Figure 7C** **)**. Since smooth muscle actin can be expressed in vascular smooth muscle cells of blood vessels and a subpopulation of interstitial fibroblasts known at the myofibroblast, protein expression was also assessed by immunohistochemistry. In normal lung, a specific smooth muscle actin (SMA) protein signal was evident in vascular smooth muscle cells of large blood vessels, and in the tips of secondary saccules, known locales of the myofibroblast [28]. In the Klf4 null lung, a specific SMA signal was only present in vascular smooth muscle cells of large blood vessels (**Figure 13**).

## Discussion

In this study, we hypothesized that changes in alveolar oxygen tension at birth could serve a physiologic function to signal expression of lung genes required for postnatal lung development. Our microarray data revealed that there is an acute change in the level of expression of 157 genes within 2 hours of birth in room air, and the number of gene changes steadily declines at 6, 12 and 24 hours thereafter. Most of the lung gene changes through 6 hours involved transient induction or repression of expression. And less than 30% of the changes involved genes in common to any two successive time points, suggesting sequential waves of new gene expression over time. Four functional categories of gene expression were overrepresented in the microarray data at 2 hours after birth in room air: transcriptional regulation, structure, apoptosis and antioxidant activity. The presence of antioxidants as a functional gene category correlates with our glutathione disulfide data, which shows the presence of transient oxidant stress in the lung at 2 hours, and suggests a response to a change in the local redox state of the lung as environmental oxygen is altered at birth.

Exposure to 10% oxygen at the end of gestation did not significantly alter baseline gene expression in the fetal lung. Nor did it adversely impact survival of fetal or newborn mice up to 24 hours after birth suggesting that changes in blood flow, lung stretch, nutrient supply and fluid resorption were similar to that of mice birthed in room air and less likely to differentially impact gene expression. Rather birth into 10% oxygen was associated with a 60% decrease in the number of gene changes at 2 hours, compared to that in room air, elimination of any overrepresented functional category of gene expression and attenuation of glutathione disulfide accumulation. Hence birth into an environment of hypoxia is associated with retention of a gene expression pattern like that of the fetal lung.

With birth into room air, there were also fewer gene expression changes in the liver at 2 hours compared to the lung, as well as a lack of overrepresentation of any functional category of gene expression. Interestingly, oxidant stress was not evident in the liver at these early time points after birth as glutathione disulfide did not accumulate. The liver may not be subject to the same magnitude of change in environmental oxygen tension as the lung or it may follow a different time course. The few gene expression levels that did change in common between these two organs could suggest a shared response to the process of birth itself. The majority of unique changes in the lung, however, suggest a lung-specific response likely related to the change in alveolar oxygen content at birth.

The gene exhibiting the greatest change in expression in the lung with birth in room air was the transcription factor Kruppel-like factor 4. Klf4, also known as gut Kruppel-like factor, was initially identified as a tissue-specific gene that inhibited cell proliferation and stimulated differentiation in the gut. The message was highly abundant in colon tissue, particularly in the epithelial cells of the crypts where there is a tight link between growth arrest and differentiation. Klf4 regulates cell proliferation by inhibiting cell cycle progression and inducing growth arrest [6]–[8]. This function is relevant in murine lung development as mesenchymal and epithelial cell proliferation is known to progressively decline starting late in gestation and continuing into the early postnatal period [29]–[31]. Transcription factors regulating this process remain poorly defined but our data suggests a role for Klf4. Hence we focused the reminder of our studies on this transcription factor. Klf4 induces growth arrest in cells via the cell cycle inhibitor p21^cip1/Waf1^ using transcriptional mechanisms to activate the p21^cip1/Waf1^ promoter and to recruit p53 to this promoter [12]. Whether this mechanism is active in the lung fibroblasts or epithelial cells will require further study, but linkage between p21^cip1/Waf1^ and Klf4 is suggested by the fact that Klf4 deficiency causes a decrease in the level of p21^cip1/Waf1^ mRNA expression in the late fetal lung. In addition, cell proliferation persists at birth in fibroblasts and airway epithelial cells.

The role of Klf4 in regulating cell differentiation is well described in the epithelium of the skin of the Klf4 null mouse [9], as well as goblet cells of the cornea [32] and the colon [25]. There is no similar description of Klf4 regulating mesenchymal cell differentiation, even though Klf4 expression has previously been described in the mesenchyme during development [6]. Our data clearly shows that connective tissue gene expression for smooth muscle actin, fibronectin, tenascin C and the alpha 1 chain of Type 1 collagen are Klf4 targets in fibroblasts of the developing lung. And in the absence of Klf4 expression, fibroblast connective tissue gene expression is down-regulated in perinatal lung. The mechanism appears transcriptional in nature, but the exact details await further study. Nonetheless, impaired connective tissue gene expression has at least two distinct biological implications for postnatal lung development. First, the newborn lung is easily ruptured under pressure as the connective tissue matrix that forms the lung scaffold is rudimentary at birth. The tensile strength that accumulates in the postnatal period is largely due to collagen synthesis and deposition [33]. Lack of tensile strength predisposes to excessive lung compliance and emphysema [34]. Second, fibronectin, type 1 collagen and tenascin C expression together with smooth muscle actin defines a specific subpopulation of lung fibroblasts known as the myofibroblast. The cells are known to localize at the tips of secondary saccules and express connective tissue proteins that may be involved with the early postnatal formation of alveoli [28], [35]–[37]. Loss of smooth muscle actin expression in the absence of Klf4 suggests impaired myofibroblast differentiation which may disrupt alveogenesis [28]. Interestingly, smooth muscle actin protein expression in the Klf4 null lung was preserved in vascular smooth muscle cells, which correlates with the known role of Klf4 as a negative regulator of smooth muscle actin gene expression in these cells [38]. Our studies show that Klf4 is a positive regulator of smooth muscle actin gene expression in lung myofibroblasts. Therefore Klf4 differentially regulates smooth muscle actin gene expression in these two lung cell types.

The presence of increased cell death in the Klf4 null lung at birth supports a recently defined role for Klf4 as an inhibitor of apoptosis [12]. Apoptosis is present even in the normal lung at birth where it is believed to play a physiologic role in postnatal lung development [39], [40]. And our microarray data identified apoptosis-related genes as an overrepresented functional gene category among the 157 genes changes that occurred at 2 hours after birth. The mechanism by which Klf4 inhibits apoptosis is not yet fully resolved, though Klf4 can block Bax, a pro-apoptotic protein, in a p53 dependent fashion, and can directly suppress p53 transcription [12]. The presence of robust induction of activated p53, p21^cip1/Waf1^ and caspase-3 in the Klf4 null lung at birth supports this inhibitory role for Klf4 in apoptosis. While the excessive degree of apoptosis in the Klf4 null lung, compared to normal lung, could contribute to loss of gene expression at birth, the absence of apoptosis in vascular smooth muscle cells within large blood vessels indicates that apoptosis was not a general phenomenon, rather it is cell specific.

**Figure 14:**
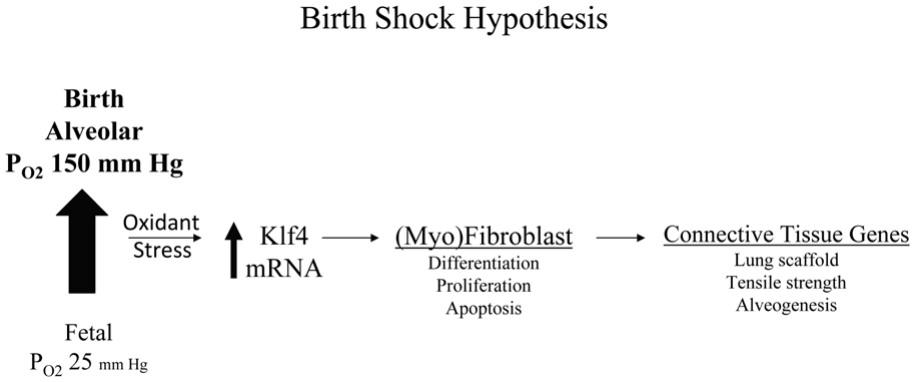
Birth shock hypothesis. We propose that the changing alveolar oxygen at birth changes the local redox state (birth shock) and thereby acts as a physiologic signal to induce Klf4 mRNA expression, which then regulates proliferation and apoptosis in fibroblasts and airway epithelial cells, and connective tissue gene expression and myofibroblast differentiation at the tips of developing alveoli.

The magnitude of apoptosis and induction of p21^cip1/Waf1^ mRNA and protein suggests that the Klf4 null lung may be under stress at birth [41]. Stress from oxidants, exposure to heat, chemical and mechanical forces, and nutrient deprivation is known to activate Klf4 expression [12]. Our data shows that Klf4 mRNA induction in lung fibroblasts exposed to hyperoxia occurs at a transcriptional level in vitro and is not dependent on protein synthesis. The mechanisms mediating this transcriptional regulation will require further study. That oxidant stress plays a role in the response of Klf4 to oxygen in vivo is suggested by the fact that Klf4 mRNA induction in normal lung at birth is associated with transient oxidant stress, and that both Klf4 mRNA induction and the degree of oxidant stress are attenuated with birth in 10% oxygen. Alveolar oxygen is already known to affect antioxidant, cytokine and transcription factor gene expression at birth [42]–[47]. Our study is the first to suggest a physiologic role for oxygen as a regulator of a transcription factor that is required for fibroblast and myofibroblast differentiation in normal postnatal lung development **(** **Figure 14** **)**.

Lastly, our Klf4 data in perinatal lung complements existing lung microarray data that found Klf4 to be induced during development [48], [49], post-pneumonectomy [50], and with exposure to cigarette smoke [51] and ozone [52]. Taken together, Klf4 regulated developmental pathways are likely recapitulated during repair of lung injury. Our study extends the role of Kruppel-like family members in lung development beyond Klf2, lung Kruppel like factor [53], [54], and Klf5 [11] and shows that Klf4 serves a distinct role in regulating lung myofibroblast connective tissue gene expression. Since the gene targeted Klf4 null mouse dies within 15 hours of birth, ultimate proof of this birth shock hypothesis awaits conditional deletion of Klf4 expression from lung fibroblasts in the perinatal period.
